# France’s New Lung Transplant Allocation System: Combining Equity With Proximity by Optimizing Geographic Boundaries Through the Supply/Demand Ratio

**DOI:** 10.3389/ti.2022.10049

**Published:** 2022-05-24

**Authors:** Florian Bayer, Richard Dorent, Christelle Cantrelle, Camille Legeai, François Kerbaul, Christian Jacquelinet

**Affiliations:** ^1^ Agence de la Biomédecine, Saint-Denis, France; ^2^ Inserm U1018, CESP, Villejuif, France

**Keywords:** geographic, organ procurment, lung transplant, allocation system, geographic disparity

## Abstract

A new lung allocation system was introduced in France in September 2020. It aimed to reduce geographic disparities in lung allocation while maintaining proximity. In the previous two-tiered priority-based system, grafts not allocated through national high-urgency status were offered to transplant centres according to geographic criteria. Between 2013 and 2018, significant geographic disparities in transplant allocation were observed across transplant centres with a mean number of grafts offered per candidate ranging from 1.4 to 5.2. The new system redistricted the local allocation units according to supply/demand ratio, removed regional sharing and increased national sharing. The supply/demand ratio was defined as the ratio of lungs recovered within the local allocation unit to transplants performed in the centre. A driving time between the procurement and transplant centres of less than 2 h was retained for proximity. Using a brute-force algorithm, we designed new local allocation units that gave a supply/demand ratio of 0.5 for all the transplant centres. Under the new system, standard-deviation of graft offers per candidate decreased from 0.9 to 0.5 (*p* = 0.08) whereas the mean distance from procurement to transplant centre did not change. These preliminary results show that a supply/demand ratio-based allocation system can achieve equity while maintaining proximity.

## GRAPHICAL ABSTRACT

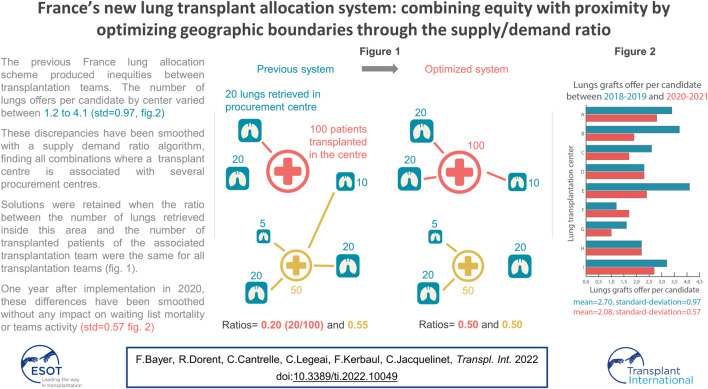

## Introduction

Geographic model in lung allocation policies varies from one country to another. In Germany, Spain, United Kingdom, United States or France, the lung allocation models combine geography with urgency. Usually, national allocation is applied for the most urgent tier and local or regional allocation for non-urgent tiers (1), with different geographic models across the tiers. In the United States, Germany and Netherlands patient’s urgency is determined with the lung allocation score (LAS). While Germany and Netherlands use the LAS for national allocation without geographic boundaries within the country, the USA applies the LAS within circular areas around the donor hospital. To the best of our knowledge, no countries use a borderless gravity model that weighs a risk score by the distance (2, 3, 4) for lung allocation. It is noteworthy that regardless of the lung allocation model and the country, the geographic component is usually defined as the distance from the procurement site or according to an administrative entity. In the United States, it has been shown that this approach is associated with disparities in graft allocation (5) and waiting list mortality (6). Broader geographic lung sharing might reduce but not resolve disparities in waiting list mortality (6). Another approach consists in defining geographical zones as a function of the population density, rather than the distance; however, this does not appear to reduce geographic disparities in the supply/demand ratio (7). It is noteworthy that the US Organ Procurement and Transplantation Network is currently developing a continuous lung graft allocation system that removes geographical boundaries. The system considers the medical urgency, the candidate’s age, the distance between the procurement and the transplantation centres, and the donor and candidate’s blood groups together (8). This quest for equity while maintaining proximity prompted the French Agence de la biomédecine to introduce in 8 September 2020 a new lung allocation system based on the supply/demand ratio. The objectives of the present study were to describe the new geographic model and show the early results.

## France’s Previous Lung Transplant Allocation System

### Background

In France there are 183 organ procurement centres (OPCs) and nine adult lung transplant centres (LTCs). In 2018, 419 patients were newly registered on the national waiting list and 373 transplants (5.5 per million population) were performed, which corresponds to 1.1 new candidates for one graft (9). Between 2013 and 2018, the 1-year cumulative incidence of transplantation estimated with competing risk analysis for newly registered candidates was 85% (83%–86%) and the 1-year cumulative incidence of death or withdrawal from the waiting list for worsening medical conditions was 6% (5%–7%) (9).

### The Previous Allocation Model

France’s allocation rules are developed by the Agence de la biomédecine, in collaboration with the transplant community. The previous two-tiered lung transplant allocation system was based on national allocation for patients with high-urgency (HU) status and local, regional, and then national allocation for elective patients.

HU status based on immediate risk of death is requested by the transplant centres and assigned by a transplant physician from another region. HU status could not be granted to candidates with chronic obstructive pulmonary disease. Lungs from donors under the age of 55 not allocated to a HU candidate are first offered to elective pediatric candidates. For elective candidates, donor lungs are allocated to LTC, with selection of the candidate at the LTC discretion.

Donor lungs are allocated to identical blood type candidates but candidates with poor access to transplant as highly sensitized patients may be assigned an exception with compatible blood type donor lung offers. For lung-kidney or lung-liver candidates, the kidney and the liver are automatically allocated with the lung.

**FIGURE 1:**
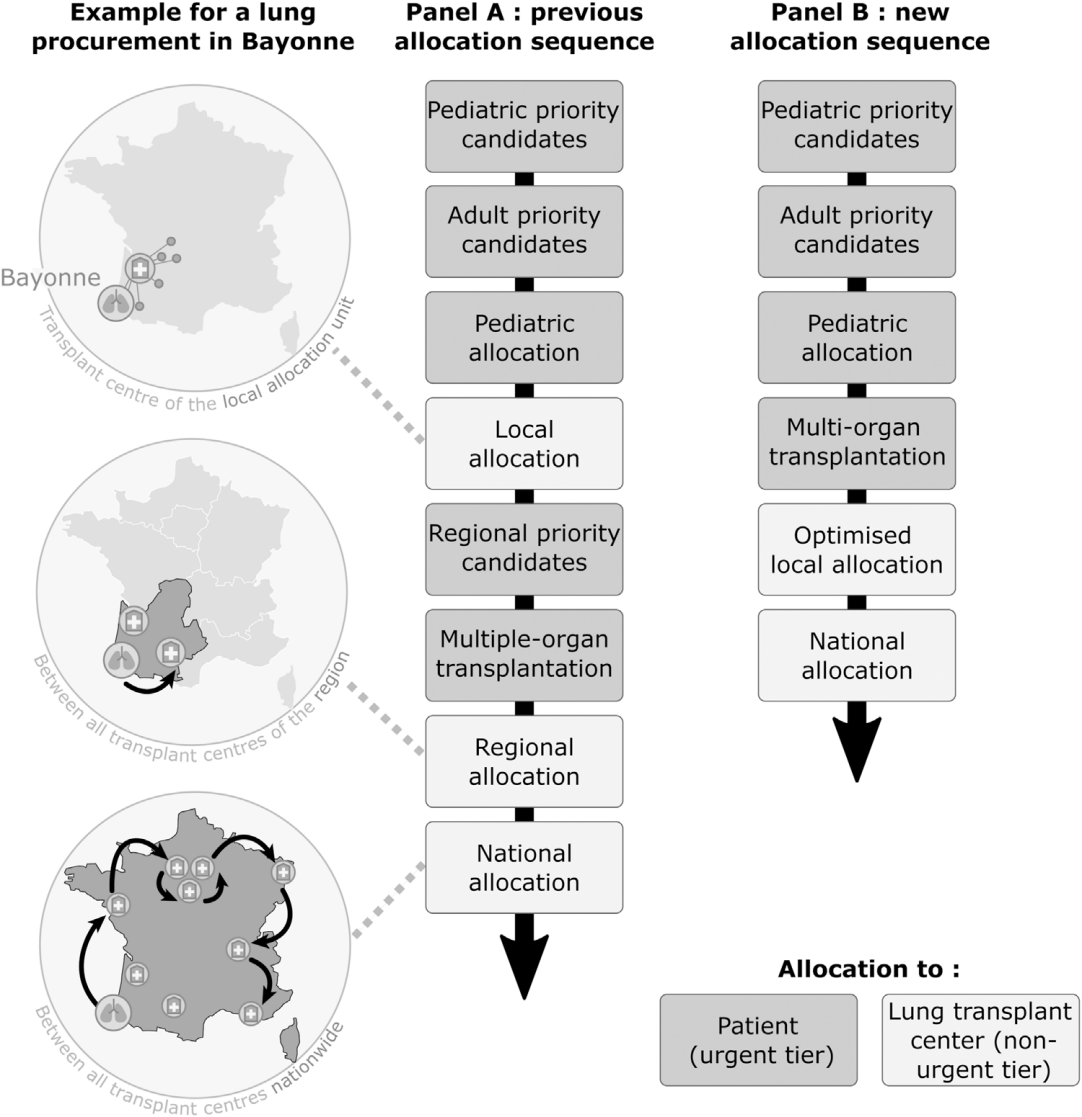
Previous **(A)** and new **(B)** lung transplant allocation sequences.

Concerning the geographical model, local allocation was based on local allocation units. One local allocation unit comprises a network of OPCs’ that serve one LTC (Figure 1, local allocation). The number of OPCs in local allocation units had been established on an historical basis. It varied from 1 to 32 between the nine French LTCs. Regional allocation was applied for donor grafts reported in OPC not part of a local allocation unit, or when the LTC, to which the OPC belonged, declined the graft and several LTC were in the same region. National allocation was applied for grafts procured in a region without LTC or when the graft was declined by the region’s LTC(s) (Figure 1A).

In 2018, 15% of grafts were allocated to HU candidates, 1% to pediatric candidates without HU status, 2% to lung-kidney and lung-liver transplant candidates, and 82% to elective adult candidates. Local, regional and national allocations accounted for 18%, 22%, and 43% of the transplants, respectively.

### The Rationale for Change

The assessment of the previous lung allocation model by the French Agence de la biomedecine identified that the sharing of brain-dead donor lungs was not fair across LTCs. Indeed, over the 2013 to 2018 period, the mean number of lungs offers per candidate, including offers to HU candidates, ranged from 1.4 to 5.2 among LTC, with a mean of 3 and a standard deviation of 1.2 (Table 1).

**TABLE 1:** Disparity in lungs sharing across transplant centres over the 2013–2018 period.

Transplant centre	Procurement hospitals in the local allocation unit (n)	Lungs recovered in the local allocation unit (n)	Candidates (n)	Lung offer per candidate (n)
Bordeaux	10	39	176	3.5
Lyon	23	17	207	4.4
Marseille	28	96	287	2.9
Marie Lannelongue	1	6	301	2.8
Nantes	32	54	153	5.2
Foch	1	21	400	1.4
Bichat	2	10	308	1.9
Strasbourg	26	72	296	2.4
Toulouse	11	19	134	3

This situation resulted from several geographic disparities. First, the number of lungs recovered from brain-dead donors differed from one region to another. Secondly, the number and medical condition of candidates differed from one LTC to another. Third, the number of OPCs within local allocation units differed among LTCs from 1 to 32, with a mean of 14.9 and a standard deviation of 12.5 (Table 1). LTCs with a larger local allocation unit have a higher probability of having a lung offer, regardless of the first two points.

These geographic disparities, together with differences in donor selection across LTCs, might account for differences in lung offers and access to transplantation across LTCs. Indeed, over the 2013–2018 period, the 1-year cumulative incidence of transplantation estimated with competing risk analysis for newly registered candidates varied from 62% (52–77) to 97% (95–99) across LTCs (9). In this context, we hypothesized that redistricting the local allocation units according to the supply/demand ratio might reduce geographic disparities while maintaining proximity-based allocation.

In addition, regional allocation had several disadvantages. Indeed, it contributed to geographic disparities in graft allocation given the number of LTCs varied from 0 to 4 across the regions and regional allocation was affecting the graft offer process to the disadvantage of national allocation.

Lastly, maintaining a prominent place for national allocation was considered as the most effective way to address geographic disparities (2).

## The New Lung Transplant Allocation System

### Materials and Methods

All data used in this study were extracted from the Cristal national database (10), which prospectively collects data on brain-dead organ donors, organ offers and transplantations.

To develop the new allocation model, all lungs from brain-dead donors transplanted between 1 January 2013 and 31 December 2018, were included. Calculations were performed using ArcGIS Network-Analyst 10.6, numpy (11) and itertools (12) libraries in Python 3.8. The 183 OPCs and the 9 LTCs were geolocalized at exact address. The travel time was estimated using a matrix based on the national French road dataset (IGN BD TOPO) and weighted by road classification, topology, population density, and land use (13).

The effect of the changes in the allocation system was assessed by comparing the 8 September 2020 to 8 September 2021, post-implementation cohort of candidates (*n* = 285) and recipients (*n* = 197) with the season-matched pre-implementation cohort of candidates (*n* = 358) and recipients (*n* = 257) (8 September 2018 to 8 September 2019). This reference cohort was chosen to avoid a baseline situation flawed by the COVID-19 pandemic (14, 15). The specific measures were type of geographic allocation, graft travel distance between OPC and LTC, cold ischemia time, number of grafts offers per candidate, 3-month cumulative incidence of transplantation, 3-month cumulative incidence of death on the waiting list or delisting for worsening medical condition, 3-month post-transplant survival. Multi-organ transplantations, transplantations with lungs used after *ex-vivo* lung perfusion, transplantations from donors after circulatory death and re-transplantations were excluded because allocation policies for those lungs are different from the standard allocation.

The three-month cumulative incidence was calculated using Fine & Gray method considering transplantation and death or delisting from the waiting list for worsening reason as competing event. Cumulative incidence of transplantation and waitlist mortality or delisting for clinical worsening were assessed with the competing risk analysis (16). Transplanted patients on these periods were included in the three-months post-transplant cohort analyses assessed with a Kaplan-Meier estimator (17). The Student’s t-test, Bartlett’s test, Levene test and z-test were used when appropriate to compare the pre- and post-implementation variables.

### Analytical Approach

We designed optimized local allocation units to achieve geographic equity (18). The objective was to ensure that all LTCs received a similar probability of transplanted brain-dead donor lungs from their local allocation unit. For each LTCs, the supply/demand ratio was calculated, over the 2013–2018 period, as the ratio of the number of donor lungs recovered and transplanted from their local allocation unit to the number of all transplants performed in the centre.

For each LTC, all possible combinations of OPCs were calculated with a brute-force algorithm to determine all possible optimized local allocation units. For each combination, we calculated the new supply/demand ratio, defined as the number of donor lungs within the optimized local allocation unit divided by the total number of transplantations performed in the LTC (Supplementary Table S1). Local allocation units comprising fewer than 5 or more than 15 OPCs were withheld. A key constraint was to maintain a driving travel time between the OPC and the LTC of no more than 2 hours. Combinations not complying with this constraint were not retained.

**FIGURE 2:**
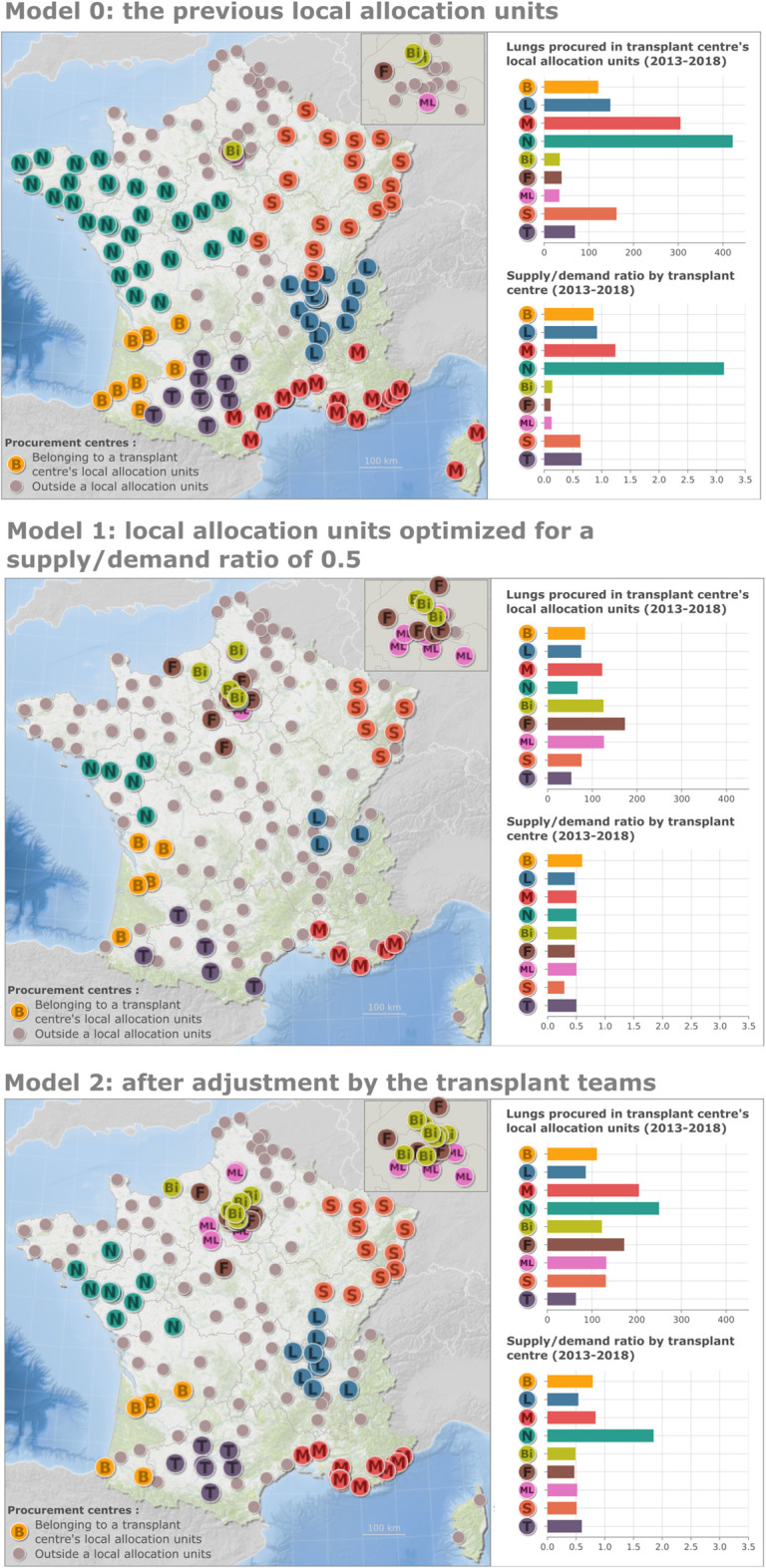
(Continued).

The judgement criteria for choice between all combinations were: 1) a similar supply/demand ratio between all LTC and 2) a lower standard deviation of the supply/demand ratio than the previous local allocation units for the nine active LTCs (Figure 2, model 0). A supply/demand ratio of 0.5 for each LTCs was selected because it ensured a lower standard deviation of the ratio among the nine LTCs compared to the previous system, while maintaining proximity between LTCs and OPCs (Figure 2, model 1). This model was tuned by LTCs that wanted to ensure that their local allocation unit still included OPCs with which they had established collaborations with regard to lung donors’ assessment and management (Figure 2, model 2).

Finally, the number of OPCs belonging to any local allocation unit decreased from 120 to 92 (Figure 2, model 2). The mean and standard deviation of the supply/demand ratio for these various geographic models are shown in Table 2.

**TABLE 2:** Mean and standard deviation for the proportion of donor lungs from the local allocation unit transplanted in the assigned transplant centre according to the geographic allocation model.

Model	Mean (*p*-value, vs. previous model)	Standard deviation (*p*-value, vs. previous model)	Min	Max
Previous model (model 0)	0.87 (ref)	0.88 (ref)	0.11	3.13
Supply/demand ratio: 0.5 (model 1)	0.48 (0.23)	0.006 (<0.001)	0.29	0.6
Final model (model 2)	0.73 (0.7)	0.19 (0.04)	0.47	1.85

In addition, the allocation sequence has been simplified by removing regional allocation (Figure 1B). OPCs not belonging to any local allocation unit are now directly offering lungs at the national level.

The developed algorithm is available online under a Creative Common license: https://github.com/fbxyz/area-optimization.

### Results

The number of candidates and transplant recipients declined respectively from 358 to 285 (−20%) and 257 to 197 (−23%) between the pre- and post-implementation periods.

Type of geographic allocation, shipping distance between OPCs and LTCs, cold ischemia time and graft offers per candidate before and after introduction of the new system are displayed in Table 3. After the new system was introduced, the percentage of transplants performed after local allocation was 20.3% vs. 25.7% before the change (*p* = 0.48). The standard deviation of the proportion of transplants performed by local share decreased across LTCs (9.8% vs. 19.1% before, *p* = 0.08). Mean and standard deviation of shipping distance slightly increased (+11 km, *p* = 0.7 and +8 km, *p* = 0.64, respectively). Mean and standard deviation of cold ischemia time decreased by 13 min (*p* = 0.08) and 7 min (*p* = 0.51), respectively.

**TABLE 3:** Lung allocation metrics for grafts from brain-dead donors before and after the change in the geographic model.

	Period		
	Pre-implementation	Post-implementation	*p*-value (Pre vs. Post)
Percentage of transplants by type of geographic allocation for each transplant centre			
mean (standard deviation)			
Local	25.7% (19.1)	20.3% (9.8)	0.48 (0.08)
Regional and national	74.3% (19.1)	79.7% (9.8)	0.48 (0.08)
Shipping distance in km for geographic allocation (Km)			
Mean	395	406	0.7
Standard deviation	296	304	0.64
Cold ischemia time			
Mean	6h11	5h58	0.08
Standard deviation	1h20	1h13	0.51
Lung offers per candidate by transplant centre			
Bordeaux	3.4	2.8	—
Lyon	3.7	1.9	—
Marseille	2.6	1.7	—
Marie Lannelongue	2.3	2.3	—
Nantes	4.1	2.5	—
Foch	1.2	1.7	—
Bichat	1.6	1	—
Strasbourg	2.2	2.2	—
Toulouse	3.2	2.7	—
Mean	2.7	2.1	0.04
Standard deviation	0.9	0.5	0.08

Under the new allocation system, mean and standard deviation of offers per candidate decreased at the edge of significance from 2.7 to 2.1 (*p* = 0.04) and 0.9 to 0.5 (*p* = 0.08).

**FIGURE 3:**
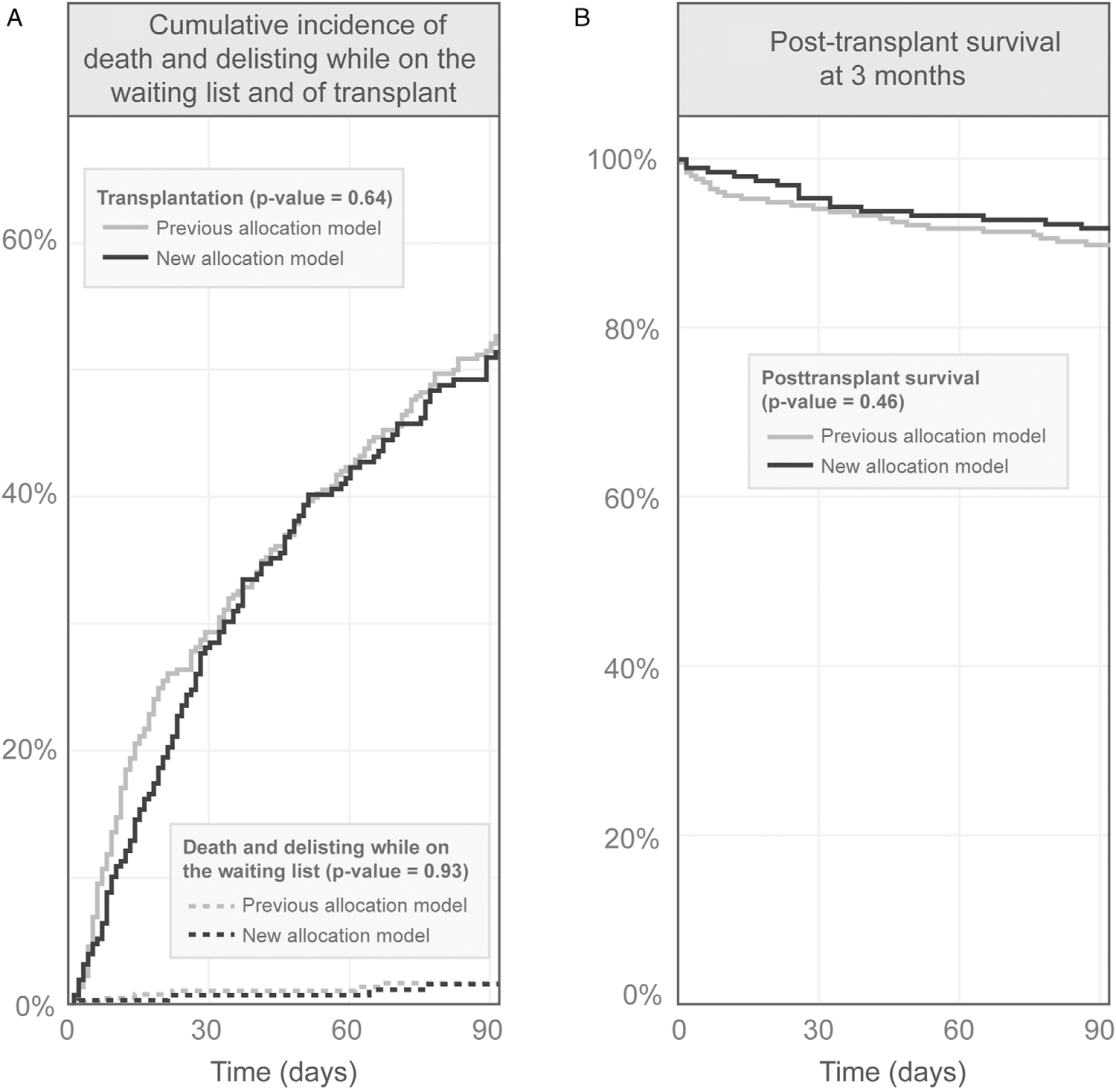
Three months survival before and after the new lung allocation system. Panel **(A)**—Cumulative incidence of death and delisting while on the waiting list and of transplant. Panel **(B)**—posttransplant survival.

The 3-month cumulative incidence of death and delisting for worsening medical condition (0.16% vs. 0.17% before, *p* = 0.93) as well as cumulative incidence of transplantation (51.5% vs. 52.3% before, *p* = 0.64) did not change between the two periods (Figure 3A). The 3-month post-transplant survival remained also unchanged (0.92 vs. 0.90 before, *p* = 0.46) (Figure 3B).

## Discussion

In September 2020, the French Agence de la biomedicine in collaboration with the transplant community implemented a new geographic lung allocation system for non-urgent candidates. The new system aimed to address geographic disparities in lung sharing through redistricting local allocation units, removing regional allocation, and improving national distribution.

While the use of LAS was discussed with support from some French transplant centres, the system has remained an urgency tier-based system, with graft allocation first to HU candidates in an immediate life-threatening situation. The LAS usefully takes account of waiting list and post-transplant mortality within 1 year (19). It has been shown that LAS-based lung allocation is associated with lower waiting list mortality, greater number of transplants, and change in the distribution of transplant indications in US (20). Furthermore, a LAS-based system allocates lungs among candidates in a transparent way without intervention from physicians. Lastly, applied as a national score with a single national waiting list, the LAS enables donor-recipient matching, which is not possible when each centre has its own waiting list. Despite these advantages, the LAS was not introduced as in most other European countries (1). This rejection can be explained by a focus on short-term patient outcome, survival benefit rather than life years gained with transplantation and lack of consideration for quality of life (1). The French system grants HU status to candidates according to therapies, namely non-invasive positive-pressure ventilation, mechanical ventilation or ECMO in patients with cystic fibrosis or bronchiectasis, high-flow non-invasive ventilation, mechanical ventilation or ECMO in patients with restrictive lung diseases, and inotropic support or use of multiple pulmonary arterial hypertension-specific drugs in patients with pulmonary vascular disease. The analysis of the 2013–2018 data showed that these HU criteria reliably predict the risk of waiting list mortality with a 3-month cumulative incidence of death or delisting for worsening medical condition of 6% (4%–8%) in HU patients and 3% (2%–4%) in non-urgent patients (9). Overall, the 10.6 deaths per 100 waitlist-years mortality rate in France in 2018 (9) compared favorably with that of US (21).

The primary intention of the new system was to reduce geographic disparities in lung supply to non-urgent candidates. Among all the reasons for such disparities, the local allocation units’ make-up was the most easy to change determinant. A brute-force algorithm was used to design new optimized local allocation units. All combinations ensuring a similar supply/demand ratio among LTCs were explored. Finally, new local allocation units were designed to provide each LTC with approximately 50% of their lung grafts demand.

We used the standard deviation of the mean number of lungs offers per candidate across the nine LTCs as a metric for equity. Potential changes in geographic disparity with the new system were not simulated before its implementation, since the geographic allocation is centre- and not patient-based. Indeed, amount of patients per transplant centre was too low and selection criteria varying a given day.

The main 1 year consequences of the implementation of the new geographic allocation system are that allocating lungs to LTCs according to supply/demand ratio reduces disparities in graft offers per candidate and disparities in percentage of transplants performed by local allocation across LTCs without increasing the distance traveled by lungs. Mean dropped significantly while standard deviation results are at the edge of significance due to the low sample number. A lower ratio of 0.05 points would make the differences significant. Not to mention that the post-implementation period was affected by the COVID-19 epidemic, which reduced transplant and lung procurement activity. Thus, the result of the modification in the allocation system aligns with the goals of the new policy. No unexpected changes in type of geographic allocation, cold ischemia time, and pre- and post-transplant outcomes were observed. Even if they are not significant, these indicators seem to improve with the new system.

The new system has several advantages over the previous French geographic allocation system and over geographic allocation systems based on fixed distance. First and most importantly the supply/demand ratio-based system can reduce geographic disparities in the number of grafts offered per candidate across LTCs. Secondly, short transport distances are maintained for a significant pool of transplants. Thirdly, the system is easy to adjust in case of local lung procurement and transplant activities change. Likewise, the local allocation units can be modified if a transplant centre opens or closes. Finally, regional allocation has now been cancelled, speeding up the lung allocation process.

The new system has also several limitations. We designed local allocation units using the number of transplants rather than the number of candidates as an index of demand. The reason was to take account of differences in graft selection among LTCs. Indeed, assessment of the previous system indicated that the rate of lung discard ranged between 34% and 85% among the nine transplant centres (data not shown). Another limitation is the modification of local allocation units by the LTCs resulting in a mean supply/demand ratio of 0.73 instead of 0.5. Indeed, the modification of the allocation system required a general acceptance of all LTCs, some of which are attached to their historical local allocation unit. An additional limitation was the algorithmic method used for the construction of the geographic model. The addition of new OPC to the model increases the calculation time in an exponential manner. There is therefore a nondeterministic polynomial time concern, like the knapsack problem (22). Without the constraints imposed on the model, the number of possible combinations would have been 10^183^, which is not feasible computationally*.* We also tested some gerrymandering algorithms (23) and knapsack algorithms. The former did not generate reproducible local allocation units close to their LTCs whereas the latter provided a single solution. Lastly, out conclusion of the new system are to be relativized because of the short period of hindsight, especially during ongoing COVID-19 pandemic.

## Conclusion

A new geographic lung allocation system based on supply/demand ratio was introduced in France in September 2020. The new system was expected to reduce geographic disparity in the number of grafts offered per candidate to non-urgent patients while maintaining proximity. The expected changes were apparent 1 year after the implementation of the new system. Long- term comprehensive monitoring of the allocation policy change is underway.

## Data Availability

The datasets presented in this article are not readily available because Data not available due to legal restrictions. Requests to access the datasets should be directed to FB, florian.bayer@biomedecine.fr.

## Abbreviations

ECMO, extracorporeal membrane oxygenation; HU, high-urgency; LAS, lung allocation score; LTC, lung transplant centre; OPC, organ procurement centre.

## References

[1] Holm AM Immer F Benden C. Lung Allocation for Transplant: The European Perspective. Clin Transpl (2020) 34(7):e13883. 10.1111/ctr.13883 32294267

[2] Bayer F Audry B Antoine C Jasseron C Legeai C Bastien O Removing Administrative Boundaries Using a Gravity Model for a National Liver Allocation System. Am J Transpl (2021) 21(3):1080–91. 10.1111/ajt.16214 32659870

[3] Jacquelinet C Audry B Bayer F Macher M-A. The New Kidney Allocation System in france Results in a Significant Increase in Transplant Access Rate, Age and Hla Dr-Qd Matching for Young Adults. Transpl Int (2017) 30:125. 10.1111/ajt.14304

[4] Dorent R Jasseron C Audry B Bayer F Legeai C Cantrelle C New French Heart Allocation System: Comparison with Eurotransplant and US Allocation Systems. Am J Transpl (2020) 20(5):1236–43. 10.1111/ajt.15816 32037718

[5] Kosztowski M Zhou S Bush E Higgins RS Segev DL Gentry SE. Geographic Disparities in Lung Transplant Rates. Am J Transpl (2019) 19(5):1491–7. 10.1111/ajt.15182 PMC6482076 30431704

[6] Mooney JJ Bhattacharya J Dhillon GS. Effect of Broader Geographic Sharing of Donor Lungs on Lung Transplant Waitlist Outcomes. J Heart Lung Transplant (2019) 38(2):136–44. 10.1016/j.healun.2018.09.007 30344025 PMC6351184

[7] Haugen CE Ishaque T Sapirstein A Cauneac A Segev DL Gentry S. Geographic Disparities in Liver Supply/demand Ratio within Fixed‐distance and Fixed‐population Circles. Am J Transpl (2019) 19(7):2044–52. 10.1111/ajt.15297 PMC6591030 30748095

[8] Stewart DE Wood DW Alcorn JB Lease ED Hayes M Hauber B A Revealed Preference Analysis to Develop Composite Scores Approximating Lung Allocation Policy in the U.S. BMC Med Inform Decis Mak (2021) 21(1):8. 10.1186/s12911-020-01377-7 33407427 PMC7789710

[9] Le Rapport Médical et Scientifique Du Prélèvement et de La Greffe En France. Agence de la biomédecine. Agence de la biomédecine - Le rapport annuel médical et scientifique 2017 (agence-biomedecine.fr). Saint-Denis: Agence de la biomédecine (2018).

[10] Strang WN Tuppin P Atinault A Jacquelinet C. The French Organ Transplant Data System. Stud Health Technol Inform (2005) 116:77–82. 16160239

[11] Harris CR Millman KJ van der Walt SJ Gommers R Virtanen P Cournapeau D Array Programming with NumPy. Nature (2020) 585(7825):357–62. 10.1038/s41586-020-2649-2 32939066 PMC7759461

[12] Lott SF. Functional Python Programming. Birmingham: Packt Publishing (2018). Available from: http://univ.scholarvox.com.proxy.scd.u-psud.fr/book/88856834 (Accessed November 18, 2020).

[13] Bayer F Le Neindre C. Mesurer l’accessibilité spatiale aux soins en France. Presented at the. Versailles: Congrès ESRI (2016).

[14] Loupy A Aubert O Reese PP Bastien O Bayer F Jacquelinet C. Organ Procurement and Transplantation during the COVID-19 Pandemic. The Lancet (2020) 395(10237):e95–e96. 10.1016/s0140-6736(20)31040-0 PMC7213957 32407668

[15] Legeai C. Impact of Coronavirus Disease 2019 on Organ Donation and Transplantation in France. Transpl Int (2020). 10.1111/tri.13769 33068462

[16] Fine JP Gray RJ. A Proportional Hazards Model for the Subdistribution of a Competing Risk. J Am Stat Assoc (1999) 94(446):496–509. 10.1080/01621459.1999.10474144

[17] Kaplan EL Meier P. Nonparametric Estimation from Incomplete Observations. J Am Stat Assoc (1958) 53(282):457–81. 10.1080/01621459.1958.10501452

[18] Marsh MT Schilling DA. Equity Measurement in Facility Location Analysis: A Review and Framework. Eur J Oper Res (1994) 74(1):1–17. 10.1016/0377-2217(94)90200-3

[19] Egan TM Murray S Bustami RT Shearon TH McCullough KP Edwards LB Development of the New Lung Allocation System in the United States. Am J Transpl (2006) 6(5p2):1212–27. 10.1111/j.1600-6143.2006.01276.x 16613597

[20] Egan TM Edwards LB. Effect of the Lung Allocation Score on Lung Transplantation in the United States. J Heart Lung Transplant (2016) 35(4):433–9. 10.1016/j.healun.2016.01.010 26922274

[21] Valapour M Lehr CJ Skeans MA Smith JM Miller E Goff R OPTN/SRTR 2019 Annual Data Report: Lung. Am J Transpl (2021) 21:441–520. 10.1111/ajt.16495 33595190

[22] Salkin HM De Kluyver CA. The Knapsack Problem: A Survey. Naval Res Logistics (1975) 22(1):127–44. 10.1002/nav.3800220110

[23] Baas K McAuliffe C. An Empirical Bayesian Framework for Assessing Partisan Bias in Redistricting Plans. p. 28. Available from: https://github.com/ColinMcAuliffe/UnburyTheLead/raw/master/EmpiricalBayes.pdf .

