# Supplementary material for: France’s New Lung Transplant Allocation System: Combining Equity With Proximity by Optimizing Geographic Boundaries Through the Supply/Demand Ratio
Source: Transpl Int. 2022 May 24;35:10049. doi: 10.3389/ti.2022.10049 (PMC9171509; doi:10.3389/ti.2022.10049)
Supplement: Supplementary file 1 [file DataSheet1.docx]

# Supplementary Material

The new local allocation unit were built iteratively by calculating all the possible non-redundant combinations between procurement sites [
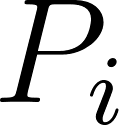
](https://www.codecogs.com/eqnedit.php?latex=P_i#0) and transplantation centres [
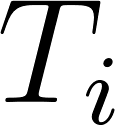
](https://www.codecogs.com/eqnedit.php?latex=T_i#0). Supplementary Table 1 gives examples of unique combinations with two transplantation centres and three procurement centres.

We then calculated the ratio [
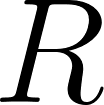
](https://www.codecogs.com/eqnedit.php?latex=R#0) between the number of lung transplants procured in each combination and the total number of transplants in the corresponding centre. For combination 1 in Table 4, for example: [
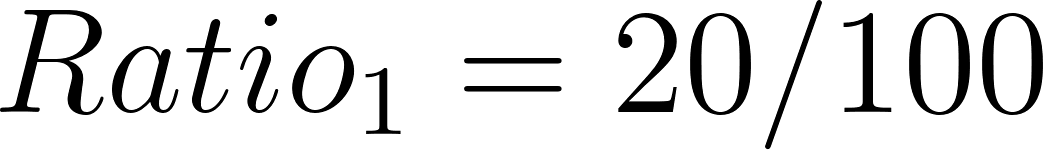
](https://www.codecogs.com/eqnedit.php?latex=Ratio_1%20%3D%2020%2F100#0) and [
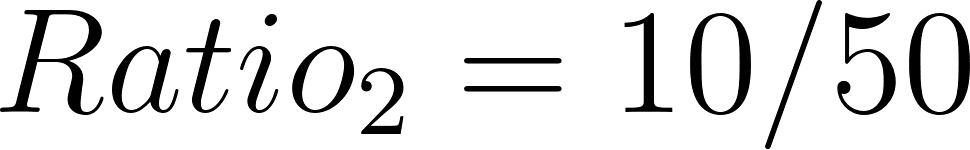
](https://www.codecogs.com/eqnedit.php?latex=Ratio_2%20%3D%2010%2F50#0). The mean and the standard deviation of the ratio is then calculated for each combination, in order to determine the model in which the mean corresponds to the target ratio and the standard deviation is lower than for other solutions. In the previous example, the mean of the two ratios for the first combination is therefore [
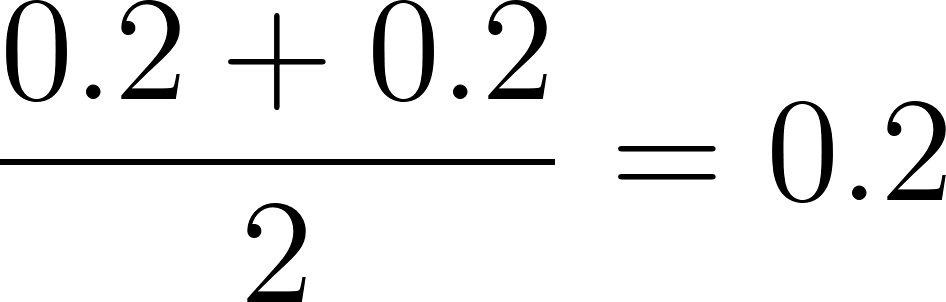
](https://www.codecogs.com/eqnedit.php?latex=%5Cfrac%7B0.2%2B0.2%7D%7B2%7D%3D0.2#0) and the standard deviation is 0, since the two means are identical.

Supplementary Table 1 - Unique combinations and calculation of the ratio

| Two transplantations teams [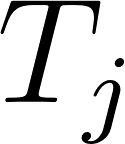](https://www.codecogs.com/eqnedit.php?latex=T_j#0) ([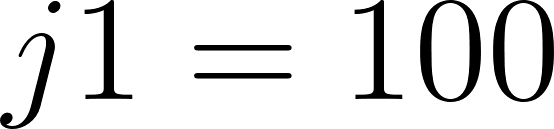](https://www.codecogs.com/eqnedit.php?latex=j1%20%3D%20100#0) transplants, [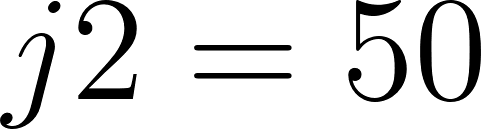](https://www.codecogs.com/eqnedit.php?latex=j2%20%3D%2050#0))  Three procurement centres [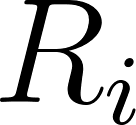](https://www.codecogs.com/eqnedit.php?latex=R_i#0) ([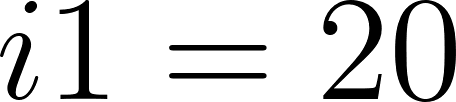](https://www.codecogs.com/eqnedit.php?latex=i1%20%3D%2020#0) lungs procured, [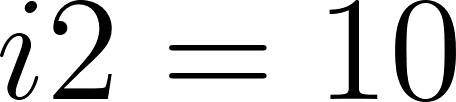](https://www.codecogs.com/eqnedit.php?latex=i2%3D10#0), [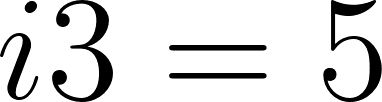](https://www.codecogs.com/eqnedit.php?latex=i3%3D5#0) | | | | |
| --- | --- | --- | --- | --- |
| All unique [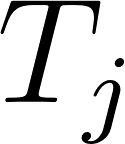](https://www.codecogs.com/eqnedit.php?latex=T_j#0) combinations | |  | [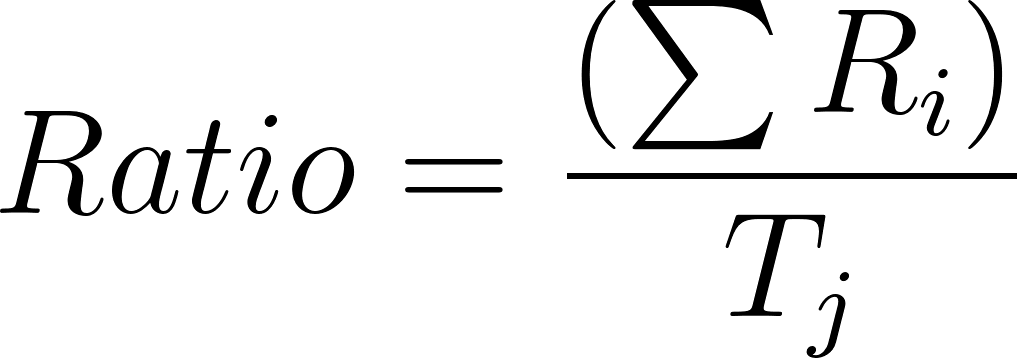](https://www.codecogs.com/eqnedit.php?latex=Ratio%20%3D%20%5Cfrac%7B(%5Csum%20R_i)%7D%7BT_j%7D#0) | |
|  |  |  | Mean | Standard deviation |
| [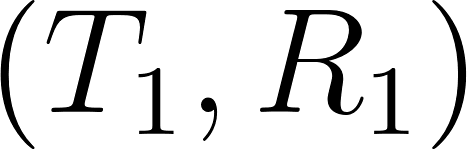](https://www.codecogs.com/eqnedit.php?latex=(T_1%2CR_1)#0) | [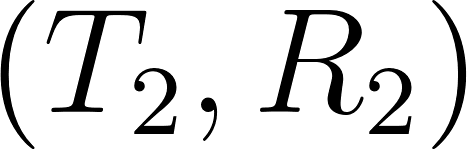](https://www.codecogs.com/eqnedit.php?latex=(T_2%2CR_2)#0) |  | (0.2 + 0.2) /2 = 0.2 | 0 |
| [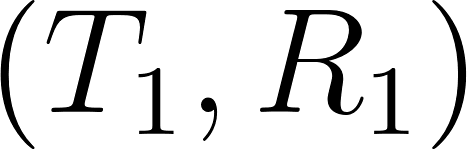](https://www.codecogs.com/eqnedit.php?latex=(T_1%2CR_1)#0) | [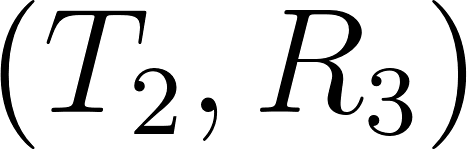](https://www.codecogs.com/eqnedit.php?latex=(T_2%2CR_3)#0) |  | 0.15 | 0.001 |
| [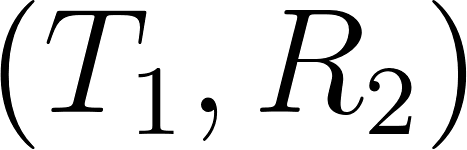](https://www.codecogs.com/eqnedit.php?latex=(T_1%2CR_2)#0) | [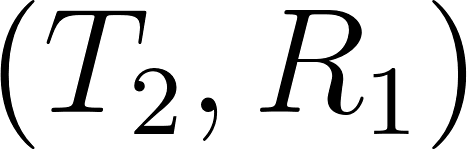](https://www.codecogs.com/eqnedit.php?latex=(T_2%2CR_1)#0) |  | 0.25 | 0.011 |
| [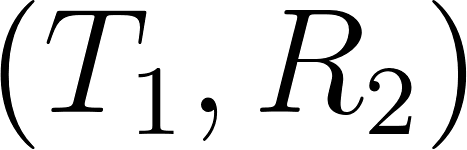](https://www.codecogs.com/eqnedit.php?latex=(T_1%2CR_2)#0) | [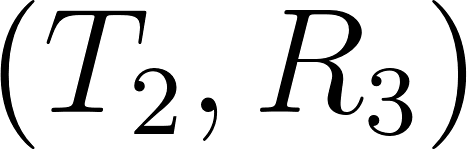](https://www.codecogs.com/eqnedit.php?latex=(T_2%2CR_3)#0) |  | 0.1 | 0 |
| [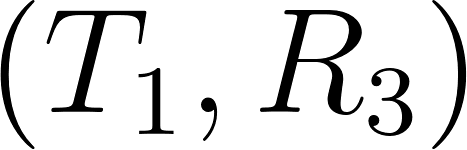](https://www.codecogs.com/eqnedit.php?latex=(T_1%2CR_3)#0) | [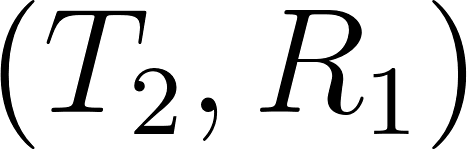](https://www.codecogs.com/eqnedit.php?latex=(T_2%2CR_1)#0) |  | 0.225 | 0.153 |
| [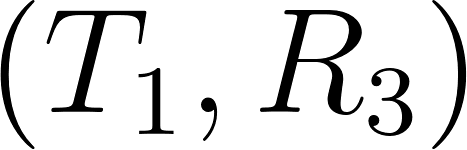](https://www.codecogs.com/eqnedit.php?latex=(T_1%2CR_3)#0) | [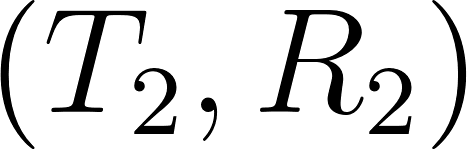](https://www.codecogs.com/eqnedit.php?latex=(T_2%2CR_2)#0) |  | 0.25 | 0.011 |
| [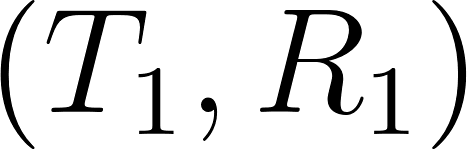](https://www.codecogs.com/eqnedit.php?latex=(T_1%2CR_1)#0) | [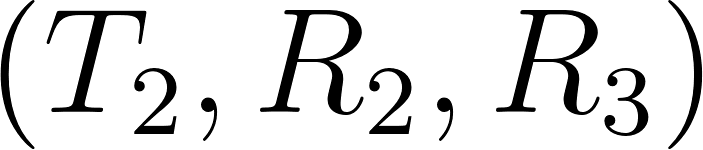](https://www.codecogs.com/eqnedit.php?latex=(T_2%2CR_2%2CR_3)#0) |  | 0.25 | 0.01 |
| [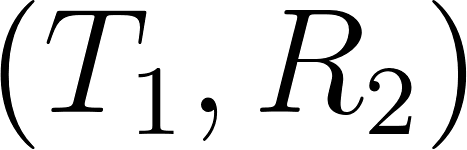](https://www.codecogs.com/eqnedit.php?latex=(T_1%2CR_2)#0) | [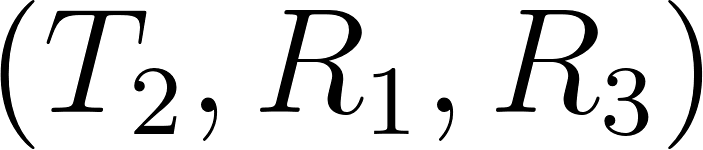](https://www.codecogs.com/eqnedit.php?latex=(T_2%2CR_1%2CR_3)#0) |  | 0.3 | 0.02 |
| [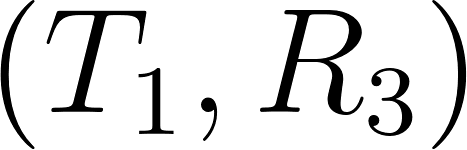](https://www.codecogs.com/eqnedit.php?latex=(T_1%2CR_3)#0) | [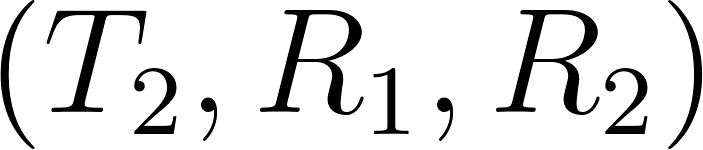](https://www.codecogs.com/eqnedit.php?latex=(T_2%2CR_1%2CR_2)#0) |  | 0.325 | 0.038 |
| [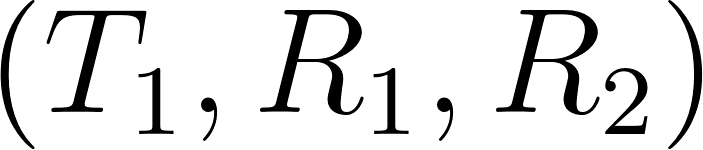](https://www.codecogs.com/eqnedit.php?latex=(T_1%2CR_1%2CR_2)#0) | [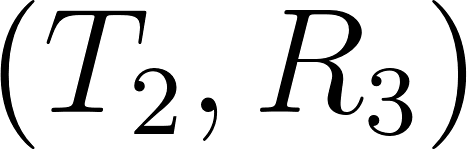](https://www.codecogs.com/eqnedit.php?latex=(T_2%2CR_3)#0) |  | 0.2 | 0.005 |
| [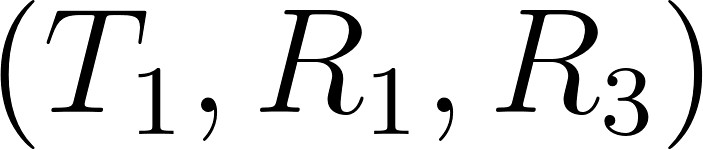](https://www.codecogs.com/eqnedit.php?latex=(T_1%2CR_1%2CR_3)#0) | [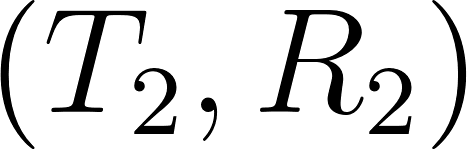](https://www.codecogs.com/eqnedit.php?latex=(T_2%2CR_2)#0) |  | 0.225 | 0 |
| [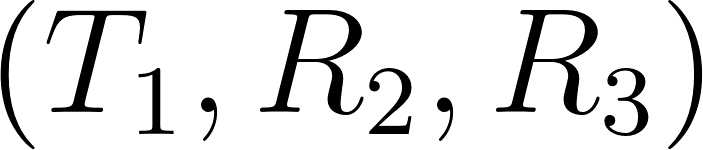](https://www.codecogs.com/eqnedit.php?latex=(T_1%2CR_2%2CR_3)#0) | [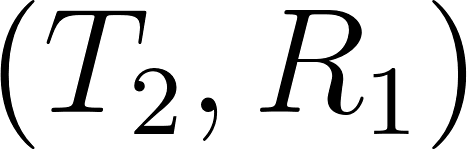](https://www.codecogs.com/eqnedit.php?latex=(T_2%2CR_1)#0) |  | 0.275 | 0.008 |
